# Lower Urinary Tract Symptom Scores Among Patients Presenting for Gender Affirming Orchiectomy: An Exploratory Analysis

**DOI:** 10.3390/ijerph23030376

**Published:** 2026-03-17

**Authors:** Jamie Michael, Vivian Wan, Kirtana Sandepudi, Sumanas Jordan, Diana K. Bowen

**Affiliations:** 1 Department of Urology, Northwestern University, Chicago, IL 60611, USA; vivian.wan@northwestern.edu (V.W.); dbowen@luriechildrens.org (D.K.B.); 2 School of Medicine, Northwestern University, Chicago, IL 60611, USA; kirtana.sandepudi@northwestern.edu; 3 Department of Plastic and Reconstructive Surgery, Northwestern University, Chicago, IL 60611, USA; sumanas.jordan@nm.org; 4 Department of Pediatric Urology, Ann and Robert Lurie Children’s Hospital, Chicago, IL 60611, USA

**Keywords:** gender-affirming care, lower urinary tract symptoms (LUTS), transgender care, patient reported outcomes, hormone therapy, mental health

## Abstract

**Highlights:**

**Public health relevance—How does this work relate to a public health issue?**

- Urinary symptoms and related distress can affect daily functioning, healthcare utilization, and quality of life in a growing transgender and gender-diverse population.
- Mental health conditions, which are highly prevalent in this population, may meaningfully shape how physical symptoms are experienced and reported.

**Public health significance—Why is this work of significance to public health?**

- This study demonstrates that symptom-related quality of life burden may be linked more to mental health history than to hormone regimen or symptom severity alone.
- These findings suggest that standard clinical metrics may underestimate patient distress in gender-diverse populations.

**Public health implications—What are the key implications or messages for practitioners, policy makers, and/or researchers in public health?**

- Multidisciplinary, gender-affirming care models should address mental health alongside urinary symptoms to improve patient-centered outcomes.
- There is a need for validated, transgender specific tools to measure urinary symptoms and related quality of life in transfeminine populations.

**Abstract:**

Current literature on baseline voiding function in gender-diverse patients assigned male at birth (AMAB) is limited. We conducted a retrospective analysis of patients presenting for gender-affirming orchiectomy consultation between 2021 and 2024 who completed the American Urological Association symptom score (AUA-SS) questionnaire to characterize baseline urinary symptoms and identify factors associated with symptom-related bother. Demographics, hormone therapy characteristics, and comorbidities were collected. Bother score was analyzed as both an ordinal variable (score 0 = “delighted” to 6 = “terrible”) and dichotomized (no bother = “delighted” or “pleased” and some bother = “mostly satisfied” to “terrible”). Twenty-six patients met the inclusion criteria, with a median age of 28 years. Overall symptom burden was low (median AUA-SS 4, IQR 2–7.3), with frequency and nocturia reported most commonly. Despite mild symptoms, most patients did not select the lowest possible bother score (“delighted”). Total AUA-SS and bother were not significantly associated with hormone therapy type or duration. In contrast, those with a documented history of anxiety, depression, or bipolar disorder had significantly higher bother score compared to those without (mean rank 15.3 vs. 9.6, *p* = 0.04). In this single-center cohort, urinary symptoms were generally mild among AMAB patients seeking gender-affirming orchiectomy. Psychiatric history was associated with a higher bother score, though this relationship was no longer significant when bother was dichotomized. These findings suggest behavioral factors may influence how urinary symptoms are experienced and reported in this population. These findings are exploratory and warrant validation in larger cohorts.

## 1. Introduction

Transgender and gender diverse (TGD) individuals often navigate unique and multifaceted healthcare challenges. More specifically, patients assigned male at birth (AMAB) may be at increased risk for baseline lower urinary tract symptoms (LUTS) due to multiple factors.

Hormone therapy, while essential for gender affirmation, may interact with the urinary system in ways that can create or worsen LUTS in this population. A large portion of transfeminine patients utilize estrogen and estrogen analogues prior to gender affirming surgery [1]. Estrogens have been shown to both promote and inhibit prostatic proliferation, potentially contributing to benign prostatic hyperplasia and LUTS [2]. In many cases, estrogen alone is not sufficient to lower serum testosterone levels to a feminine level [3]. Spironolactone is often added to estrogen medications to lower or block the effects of testosterone [4,5]. While spironolactone is an anti-androgen, it is also a diuretic, which may contribute to LUTS in this population [6].

Furthermore, transgender individuals are particularly vulnerable to psychological distress. Higher rates of anxiety and depression have been demonstrated in this group compared to the general population [7,8]. A strong correlation has been made previously between LUTS and the development of anxiety [9]. However, research also shows this relationship may be bidirectional, representing another possible contributor to LUTS in the TGD population [10]. Finally, TGD individuals may experience LUTS due to social factors such as holding behaviors associated with avoiding bathrooms [11].

There has been research regarding urinary symptoms following gender affirming surgery [12,13]. However, limited research exists exploring baseline urinary symptoms among AMAB patients before surgery. Prior studies suggest patients feel frustrated about the lack of education from providers regarding potential urinary side effects of hormone therapy [14]. The aim of this study was to characterize baseline LUTS and identify factors associated with symptom-related bother in AMAB patients presenting for gender-affirming orchiectomy consultation. In this study, we found that urinary symptom-related bother was more strongly associated with mental health history than with hormone regimen or duration.

## 2. Materials and Methods

This study received institutional review board approval by Northwestern University (STU00213075, approved 9 September 2023). This was a retrospective study utilizing deidentified data, and patient consent was not required.

### 2.1. Study Design and Patient Population

This was a single-site cohort study conducted at a tertiary academic care center with a multidisciplinary clinic serving transgender and gender diverse patients. Gender affirming orchiectomy consultation encounters with a single provider (DKB) between January 2021 and October 2024 were systematically reviewed. Patients were included if they completed an AUA-SS questionnaire, which is routinely administered at surgical consultations in our institution’s clinic [15]. Standardized administration of the AUA-SS questionnaire as part of routine clinical evaluation began in January 2021; therefore, earlier patients were not eligible for inclusion due to a lack of systematically collected questionnaire data. Of the 51 eligible patients seen during the study period, 26 (51%) had a completed AUA-SS questionnaire available for analysis. The remaining 25 patients did not have a questionnaire on record. The specific reason for non-completion could not be determined from retrospective chart review, but likely reflects real-world clinical factors such as patient refusal or time constraints. For analyses involving the AUA-SS bother score, complete-case analysis was performed. Patients missing the bother item were excluded from the primary outcome analysis. No imputation was performed due to the small sample size. To address potential responder bias, we compared patients who completed the AUA-SS with those who did not on available demographic and clinical characteristics.

### 2.2. Variables of Interest

Demographic variables, including age, body mass index (BMI), race, ethnicity, and gender, were collected. Clinical data related to gender affirmation, including smoking status, hormone therapy (HT) type, and duration, were collected. Additional variables were collected, including comorbidities and a history of urinary tract infections (UTIs). Data were electronically entered into a deidentified research database at our institution.

### 2.3. Measures

Our primary outcome was urinary symptom-related quality of life, evaluated using the AUA-SS bother score, a single-item measure that asks patients how they would feel if they had to live the rest of their life with their current urinary symptoms. Responses range from 0 (‘delighted’) to 6 (‘terrible’), with higher scores indicating greater bother. Bother was chosen over total symptom score for stratification because it reflects the subjective impact of symptoms on quality of life, which may differ between patients with similar symptom severity.

Given our small sample size (n = 25 with complete AUA-SS data), the bother score was analyzed primarily as an ordinal outcome using the Mann–Whitney U test. A sensitivity analysis dichotomized bother into no bother (delighted + pleased) versus some bother (mostly satisfied to terrible) to reflect a clinically meaningful threshold.

Due to the limited sample size, multivariable modeling was not performed. Associations are presented as unadjusted and should be interpreted as exploratory.

### 2.4. Statistical Analysis

Categorical variables were presented as percentages, and continuous variables were presented as medians (interquartile range [IQR]). Responders were compared to non-responders using the Mann–Whitney U test for continuous variables and chi-square or Fisher’s exact test for categorical variables. Bother score was analyzed as an ordinal outcome using the Mann–Whitney U test. Associations between baseline characteristics and bother group (no bother vs. some bother) were assessed using the Mann–Whitney U test for continuous variables and Fisher’s exact test for categorical variables. To quantify effect sizes for Mann–Whitney comparisons, rank-biserial correlation (r_rb) was calculated, providing a measure of the magnitude and direction of differences between groups. Given the exploratory nature and small sample size, analyses are presented using medians [IQR], mean ranks, and rank–biserial correlation rather than confidence intervals, which may be unstable in very small samples. Statistical significance was set at a two-sided alpha of 0.05. Data were analyzed using StataSE v18.0 (StataCorp, 2020. College Station, TX, USA) statistical software. The statistical code used for analyses and de-identified data are available from the corresponding author upon request.

## 3. Results

### 3.1. Study Population

We identified 51 AMAB patients who presented for gender-affirming orchiectomy consultation, of whom 26 completed the AUA-SS questionnaire. Compared to patients who completed questionnaires, those who did not were more likely to have missing gender identity on chart review (either unknown or “not answered”) (8% vs. 3.8%, *p* = 0.008) and have a history of obesity (44% vs. 19%, *p* = 0.004). Otherwise, groups were similar (Table 1).

Among those who completed questionnaires, the median age was 28 years (IQR 23.9, 34.6), and the median BMI was 26.7 kg/m^2^ (IQR 22.1, 35.6). The majority of patients identified as female (n = 24, 92.3%), were white (n = 22, 84.6%), and not Hispanic (n = 19, 73.1%). A majority of patients were non-smokers (n = 15, 57.7%), followed by former smokers (n = 9, 34.6%).

All patients, excluding one, were on hormone therapy (n = 25, 96.2%) for a median duration of 53.5 months (IQR 20.7, 72.8). The most common hormone therapy was estrogen analogues (n = 24, 92.3%), followed by spironolactone (n = 14, 53.8%), progesterone analogues (n = 8, 30.8%), and 5a reductase inhibitors (n = 8, 30.8%). Most patients had no history of UTIs (n = 24, 92.3%). Comorbidities most commonly represented were mood and anxiety disorders (n = 16, 61.5%), ADHD (n = 5, 19.2%), and eating disorders (n = 4, 15.4%).

### 3.2. AUA-SS Distribution and Bother Scores

**Figure 1:**
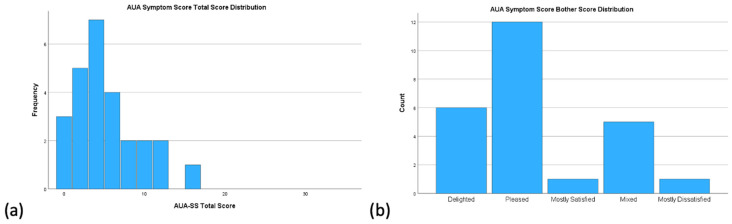
Histogram depiction of AUA symptom score results for total cohort; (**a**) AUA symptom score total score distribution, (**b**) AUA symptom score bother score distribution.

The median total AUA-SS score was 4 (IQR 2, 7.3) across all participants, which corresponds to the mild symptom category (score 0–7). Patients most commonly reported symptoms of frequency and nocturia on questionnaires, compared with other individual symptoms. Although symptom scores were low, a majority of patients recorded they were “pleased” (n = 12, 46.2%) rather than “delighted” (n = 6, 23.1%) with their symptoms. Some patients reported they were “mixed” (n = 5, 19.2%) or “mostly dissatisfied” (n = 1, 3.8%) (Figure 1).

### 3.3. Bother Groups by Baseline Characteristics

While total symptom scores were generally low, ordinal analysis of bother scores demonstrated differences associated with psychiatric history. Patients with a history of anxiety, depression, or bipolar disorder had significantly higher bother ranks compared to those without such a history (mean rank 15.3 vs. 9.6, *p* = 0.04, rank-biserial *r* = 0.45). Ordinal analysis did not demonstrate differences associated with hormone therapy type (Table 2).

In sensitivity analysis using a clinically meaningful binary cutoff, the association between psychiatric history and bother was no longer statistically significant (56% no bother vs. 71% some bother, *p* = 0.40), although the direction of effect remained similar. No associations were observed between bother and age, BMI, smoking status, hormone therapy type, or duration (Table 3).

### 3.4. Total AUA-SS Score by Hormone Therapy Type

Total AUA-SS scores were not significantly associated with the type of hormone therapy used (Supplemental Table S1). However, given the small sample size, the study was not powered to detect modest associations.

## 4. Discussion

Gender diverse AMAB patients demonstrate mild LUTS predominantly in the form of frequency and nocturia. Urinary symptom scores were not associated with hormone use. When analyzed using the full AUA-SS bother scale, psychiatric history was associated with higher reported bother. However, this association was attenuated in binary sensitivity analyses, suggesting a modest and exploratory relationship. Very few studies have investigated baseline urinary function in TGD AMAB individuals. Our exploratory analysis of voiding symptoms among patients presenting for gender affirming orchiectomy consultation raises the possibility that psychological context may influence how urinary symptoms are experienced and reported.

Prior studies investigating LUTS in this population have shown mixed results. Jiang et al. conducted a study in 2019 that sought to investigate both baseline pelvic floor dysfunction as well as outcomes of pelvic floor physical therapy in preoperative transgender females. Of their 72 patients, they identified a high incidence of urinary dysfunction (43%) at preoperative visits. On secondary analysis, urinary dysfunction was not significantly associated with reported history of abuse [16]. This study differed from ours because urinary dysfunction was diagnosed by physical therapists. In contrast, in our study, LUTS were assessed with a patient-reported questionnaire. A second study published in 2021 by Chung et al. aimed to describe genitourinary and sexual symptoms in transfeminine individuals through interviews. Of 25 transfeminine individuals assigned male at birth, 12 had undergone genital gender affirming surgery. Researchers found that patients both with and without a history of hormone therapy or gender affirming surgery reported genitourinary symptoms, including urge incontinence, stress incontinence, recurrent urinary tract and prostate infections [14]. In contrast, our study found that transfeminine patients more commonly report urinary frequency and nocturia.

We theorized that LUTS would be associated with the type or duration of hormone therapy. First, spironolactone is a diuretic, and diuretics have previously been shown to be associated with higher rates of nocturia [17]. Furthermore, animal models have demonstrated that a higher estrogen-to-androgen ratio induces LUTS and benign prostatic hyperplasia [2]. However, this relationship remains understudied. Despite this, we observed that neither total symptom score nor bother were significantly associated with duration or type of hormone therapy, though the study was not powered to detect modest effects.

One prior study has investigated the relationship between gender affirming hormone therapy and LUTS among transgender individuals. Researchers investigated 47 transgender women prospectively and gathered AUA-SS before and during gender affirming hormone therapy. Similar to our findings, they found no statistically significant changes in LUTS during treatment with estrogen preparations, except for a slight worsening in nocturia (*p* = 0.009) [18]. While this study did report baseline depression/anxiety among participants, it differs from our study as they did not assess the relationship between mood and anxiety disorders and LUTS. Despite findings from this study and ours, proper counseling about the potential impact of hormone therapy on urinary symptoms is vital. In a qualitative survey of transfeminine individuals, patients reported feeling frustrated that they had to learn about urinary side effects through their own research, rather than through education from a provider [14].

In our population, patients with a history of mood or anxiety disorders demonstrated higher bother scores. These findings suggest that psychiatric history may be associated with differences in symptom perception or appraisal, rather than objective urinary severity. Previous studies have suggested that anxiety disorders may be associated with irritative urinary symptoms [19,20,21]. In mice models, researchers previously found that repeated exposure to stress produced an overactive bladder phenotype, confirmed by increased voiding frequency and detrusor overactivity [22]. Furthermore, studies have found that there may be a dose-dependent relationship between the severity of anxiety and the irritative bladder symptoms [19]. In addition, TGD people may avoid public bathrooms due to negative experiences or associations. Holding behaviors can not only impact the urinary system physiologically but have also been associated with poorer mental health and higher suicide rates, underscoring the need for inclusive public restrooms [11]. These relationships suggest that taking care of TGD patients within the framework of a multidisciplinary clinic is beneficial so that their mental health needs can be addressed in concert with urologic care.

Importantly, the observed relationship between psychiatric history and bother was present when preserving the full ordinal scale but was attenuated when using a clinically defined binary classification. This suggests that differences may reflect graduations in symptom appraisal rather than clear distinctions between absence or presence of distress. Given known associations between anxiety traits and response style [23], these findings may reflect differences in symptom perception or reporting rather than underlying urinary pathology.

Providers should consider these dynamics as they provide care for more gender diverse patients. One potential treatment option for TGD patients presenting with baseline LUTS is pelvic floor physical therapy (PT). This can be utilized pre- and post-op to assess baseline voiding and defecation patterns and assess pelvic floor muscle tone, contraction, and relaxation. Jiang et al. found that pelvic floor PT significantly reduced rates of pelvic floor dysfunction in those who attended both preoperatively and postoperatively [16]. Additionally, there is a need for the development of trans-specific questionnaires addressing voiding and sexual health. The incontinence questionnaire-lower urinary tract symptoms (ICIQ-LUTS) questionnaire was previously validated for transmasculine patients. Future research should be aimed at validating questionnaires for transfeminine patients [24].

This study includes 26 participants with complete AUA-SS data from a single center. The limited sample size reduces statistical power and increases the risk of type II error. As such, null findings should be interpreted cautiously. However, the sample size is similar to that of other studies evaluating voiding function in this population. In addition to being from a single center, our cohort was predominantly white (84.6%), which limits the generalizability of our findings. Further, the AUA-SS is not validated in this population, and results should be interpreted with caution until validated tools are available. However, there are no validated LUTS questionnaires for trans-feminine pre-surgical patients, and AUA-SS was selected because it is routinely administered at our institution’s gender-affirming surgery clinic. Previous literature has also utilized the AUA-SS for this population [12,18]. Development of trans-specific urinary symptom questionnaires is an important priority for future research.

The interpretation of the bother score is sensitive to how the scale is categorized. While ordinal analysis demonstrated an association between psychiatric history and higher bother ranks, binary sensitivity analyses were not statistically significant, although the effect direction was similar. These findings may reflect limited statistical power, response-style differences, residual confounding (age, BMI, smoking, hormone therapy type and duration, psychotropic medications), or true modest associations. Finally, we use the AUA-SS as a correlate for true voiding function. However, the bother score component allows us to assess patient-reported quality of life. Larger studies using validated instruments and multivariable modeling are needed to clarify these relationships.

## 5. Conclusions

In this single-center cohort of AMAB patients presenting for gender-affirming orchiectomy, AUA-SS were generally low. Urinary symptom severity and symptom-related quality of life scores were not associated with hormone therapy type or duration, although this study was underpowered to detect modest effects. Psychiatric history was associated with higher bother scores when analyzed using the full ordinal scale, though this association was not statistically significant when bother was dichotomized. These findings suggest that psychological context may influence how urinary symptoms are perceived and reported in this population. Larger studies using validated instruments and multivariable modeling are needed to confirm these exploratory findings.

## Figures and Tables

**Table 1:** Patient Demographics for those who completed AUA-SS and those who did not.

Variable	Completed Questionnaire (n = 26)	Did Not Complete Questionnaire (n = 25)	*p*-Value
Age, median (IQR)	28.4 (23.9, 34.6)	33 (28.5, 41)	0.11
BMI, median (IQR)	26.7 (22.1, 35.6)	31.7 (25, 38.1)	0.11
Gender, n (%)			0.008
Trans Female	24 (92.3)	21 (84)	
Non-binary	1 (3.8)	2 (8)	
Unknown/not-answered	1 (3.8)	2 (8)	
Ethnicity, n (%) *			0.7
Not Hispanic	19 (73.1)	21 (84)	
Hispanic	5 (19.2)	2 (8)	
Smoker, n (%)			0.51
Never	15 (57.7)	14 (56)	
Former	9 (34.6)	0	
Current	2 (7.7)	11 (44)	
Hormone therapy, n (%)			1.0
Yes	25 (96.2)	25 (100)	
No	1 (3.8)	0	
Currently taking estrogen analogues, n (%)	24 (92.3)	25 (100)	0.49
Currently taking progesterone analogues, n (%)	8 (30.8)	14 (56)	0.09
Currently taking spironolactone, n (%)	14 (53.8)	15 (60)	0.78
Currently taking -5-alpha reductase inhibitor, n (%)	8 (30.8)	3 (12)	0.44
Time on HT in months, median (IQR)	53.5 (20.7, 72.8)	33.2 (27.1, 73)	0.28
History of UTIs, n (%)			0.49
No	24 (92.3)	25 (100)	
Yes	2 (7.7)	0	
History of anxiety or mood disorders, n (%)	16 (61.5)	17 (68)	0.77
History of asthma, n (%)	2 (7.7)	3 (11.5)	1.0
History of obesity, n (%)	5 (19.2)	11 (44)	0.004
History of ADHD, n (%)	2 (7.7)	4 (16)	1.0
History of PTSD, n (%)	2 (7.7)	4 (16)	0.42
History of Eating disorder, n (%)	4 (15.4)	0	0.11

* *percentages may not sum up to 100% due to missing data.*

**Table 2:** Ordinal comparison of AUA-SS bother scores by baseline characteristics.

Variable	Group	n	Mean Rank	r (Rank-Biserial)	*p*-Value
Estrogen analogues	Yes No	23 2	13.0 12.5	0.04	0.91
Progesterone analogues	Yes No	8 17	13.1 12.9	0.01	0.95
Spironolactone	Yes No	14 11	12.6 13.5	0.06	0.77
History of anxiety/ mood disorder	Yes No	15 10	15.3 9.6	0.45	0.04
Smoker (ever vs. never)	Yes No	11 14	15.1 11.3	0.31	0.17

**Table 3:** Patient characteristics by AUA Symptom Score bother group (No bother = “delighted” or “pleased”, some bother = “mostly satisfied” through “terrible”).

Variable	No Bother (n = 18) *	Some Bother (n = 7) *	*p*-Value
Age (years), median (IQR)	27 (25, 32)	32 (18, 44)	0.88
BMI (kg/m^2^), median (IQR)	26 (22, 36)	29 (25, 36)	0.68
Smoker, n (%)			0.53
Never	11 (61%)	3 (43%)	
Former/Current	7 (39%)	4 (57%)	
Estrogen analogues, n (%)			0.51
Yes	16 (89%)	7 (100%)	
No	2 (11%)	0	
Progesterone analogues, n (%)			0.16
Yes	4 (22%)	4 (57%)	
No	14 (78%)	3 (43%)	
Spironolactone, n (%)			0.65
Yes	10 (56%)	4 (57%)	
No	8 (44%)	3 (43%)	
Time on HT (months), median (IQR)	54 (13, 72)	61 (31, 276)	0.24
History of UTIs, n (%)			0.07
Yes	0	2 (29%)	
No	18 (100%)	5 (71%)	
History of Anxiety/Depression, n (%)			0.40
Yes	10 (56%)	5 (71%)	
No	8 (44%)	2 (29%)	

* *Bother score missing for one participant (n = 1); analyses include 25 patients.*

## Data Availability

The deidentified dataset used for this study is available from the corresponding author upon request.

## Supplementary Materials

The following supporting information can be downloaded at: https://www.mdpi.com/article/10.3390/ijerph23030376/s1, Table S1: AUA-SS median total score by type of hormone therapy.

## Abbreviations

The following abbreviations are used in this manuscript:

TGD	Transgender and gender diverse
AMAB	Assigned male at birth
LUTS	Lower urinary tract symptoms
AUA-SS	American Urological Association Symptom Score
BMI	Body mass index
UTIs	Urinary tract infections
HT	Hormone therapy
IQR	Interquartile range
PT	Physical therapy
