# Supplementary material for: Lower Urinary Tract Symptom Scores Among Patients Presenting for Gender Affirming Orchiectomy: An Exploratory Analysis
Source: Int J Environ Res Public Health. 2026 Mar 17;23(3):376. doi: 10.3390/ijerph23030376 (PMC13027255; doi:10.3390/ijerph23030376)
Supplement: Supplementary file 1 [file ijerph-23-00376-s001.zip › ijerph-4161369-supplementary.pdf]

Supplemental Table S1: AUA-SS median total score by type of hormone therapy

Medication	Median AUA-SS	p-value
Spironolactone		
Yes	5.0	0.10
No	4.6	
Estrogen Analogue		
Yes	4.92	0.34
No	3.50	
Progesterone Analogue		
Yes	5.38	0.16
No	4.56	
5a reductase inhibitor		
Yes	4.80	0.44
No	4.81	
